# Study protocol for a randomised controlled trial of dance-movement therapy for adolescents with depression

**DOI:** 10.1186/s40359-025-03917-0

**Published:** 2026-01-09

**Authors:** Riikka Valjakka, Päivi Pylvänäinen, Maj Lindgren, Anita Forsblom, Mauri Marttunen, Henna Haravuori

**Affiliations:** 1 Department of Psychiatry, Adolescent outpatient unit, University of Helsinki and Helsinki University hospital, P.O. Box 356,, Helsinki, 00520 Finland; 2 Tampere University Hospital, Tampere, Finland; 3 Wellbeing services County of Lapland, Rovaniemi, Finland; 4 Mental Health Team, Finnish Institute for Health and Welfare, Helsinki, Finland; 5 Adolescent Psychiatry, University of Helsinki and Helsinki University Hospital, Helsinki, Finland; 6 Vantaa, Finland

**Keywords:** Depression, Adolescents, Youth, Dance-movement therapy, Group therapy, Body image

## Abstract

**Background:**

The number of adolescents with depression has increased in mental health services over the past decade. New intervention options are needed for these patients.

**Method:**

Patients aged 13 to 17 years with depression are recruited from adolescent mental health services. Participants will be randomized into two groups. The treatment group will attend dance movement therapy for 12 weeks in addition to treatment as usual, while the control group will receive treatment as usual. The outcome measures include questionnaires and interviews assessing depression symptoms and body image. The primary outcome measures are the Beck Depression Inventory (BDI-21) and the Young Person’s Clinical Outcome in Routine Evaluation (YP-CORE). Additional measures include the Adolescent Depression Rating Scale (ADRS) and a body image scale.

**Discussion:**

This study will provide information about the effectiveness of dance-movement therapy for adolescents with depression in a naturalistic environment. Variety in the choice of therapies may aid in quicker improvement and prevent prolonged depression.

**Trial registration:**

This protocol was registrated 29.8.2022 at ClinicalTrials.gov. with identifier. Clinical trial number 07293806.

## Background

Depression is a common psychiatric disorder and a significant general health problem among adolescents [1]. The peak incidence of depression occurs between 15 and 18 years of age, and by adulthood, 15% of individuals have experienced depression [2]. The prevalence of mild to severe depressive symptoms in children and adolescents is 21.3% and has increased over time [3]. Depression during early years is a disabling health problem with a high risk for recurrence [1, 4].

Approximately one-third of young patients with depression receiving current treatments, such as talk-based psychotherapies and antidepressant medication do not recover [5, 6]. Additional research on depression and the development of new treatment options are needed to improve outcomes in depression care for youth. Special attention should be focused on young patients with depression, as early interventions are most effective [1].

### Exercise for depression

Numerous studies have demonstrated that physical training is beneficial in the treatment of depression. Aerobic exercise can significantly improve depressive symptoms in young individuals, with those having major depressive disorder (MDD) benefiting the most. Even low-intensity exercise can alleviate depressive symptoms, although outcomes are more favorable with moderate-intensity exercise. Physical activity is a recommended intervention for depression [7, 8].

However, initiating physical training can be challenging for adolescents with depression. Many symptoms of depression are related to the body, including fatigue, exhaustion, psychomotor retardation, loss of appetite, self-injury, and self-criticism or self-disgust, often directed toward one’s own body. Depression symptoms are sometimes accompanied by somatic symptoms, such as bodily pain. Given the relationship between depression and bodily experience, the question arises as to whether depression can be treated with therapy based on body-oriented methods [9].

Dance may serve as a feasible alternative to traditional physical activity. A systematic review demonstrates that dance provides physiological and psychological benefits to both healthy and medically compromised populations [10].

### Dance-movement therapy

Dance Movement Therapy (DMT) is based on the body-mind connection, where emotions are represented in the body as physical sensations and reactions [11, 12]. In DMT, body movement serves both as a means of expression and as a therapeutic tool [13]. DMT utilizes knowledge from various dance forms, improvisational and expressive movement, and somatic techniques [14].

Like other creative therapies, the effects of dance movement therapy (DMT) can be divided into five categories: (1) experience of pleasure and playfulness, (2) experience and communication of aesthetics, (3) nonverbal communication, (4) experience of activeness and independence, and (5) creativity [11]. As an exercise and action-based therapy, DMT is assumed to also involve the benefits of exercise, such as balancing the autonomic nervous system and hormonal changes [15, 16]. A dance-related specific effect is the cultivation of proprioceptive and interoceptive senses, which build body awareness [13]. A correlation exists between the development of interoception and sensitivity to emotions and empathy [13]. Furthermore, general effects of psychotherapies, such as the therapeutic relationship, and group-therapy-related phenomena, like group cohesion and peer support, contribute to the effects of DMT [11].

The origins of dance movement therapy (DMT) are rooted in psychodynamic theory, but its methods incorporate theories from various psychotherapies. DMT combines exercise, creativity, and cognitive and social components [13]. The clinical relevance of playfulness and creativity is based on acceptance and safe relationships for communication, which promote social relating and prosocial behavior [17]. Body movement can be symbolic and allows for an alternative mode of communication. Nonverbal communication through movement can facilitate verbal communication as well [17].

### Dance-movement therapy for depression

Dance-movement therapy (DMT) is a promising treatment for depression among adult patients. A systematic review and meta-analysis of eight studies included 351 patients with depression, of whom 192 received DMT combined with treatment as usual (TAU) and 159 received TAU only. All studies showed a decrease in the severity of depression. One study in the meta-analysis reported moderate depression symptoms decreasing to minimal symptoms. The effect size of DMT was 0.64 compared to control groups. The meta-analysis concluded that DMT is a beneficial intervention in the treatment of depression. However, only one study in the review included young patients, indicating a need for future studies, especially targeting this population [18].

Research on DMT in the treatment of adolescent depression is limited. A Korean trial assessed the effectiveness of DMT for mild depression in young patients, with a mean age of 16 years. Patients were randomized to either a DMT group or a waiting list control group, with 20 patients in each group. In the DMT group, depressive symptoms decreased compared to the control group. Additionally, participants in the DMT group showed an increase in plasma serotonin concentration and a decrease in dopamine concentration compared to the control group [19].

In a Chinese study, 62 adolescents with depression were divided into experimental and control groups. The experimental group (32 participants) received group psychological intervention and dance therapy based on the Satir Model, whereas the control group (30 participants) did not receive any intervention. The depression and anxiety levels in the experimental group were significantly lower than those in the control group. The combination of group intervention and dance therapy based on the Satir Model was found to be a feasible method to alleviate anxiety and depression in adolescents and to promote life satisfaction and resilience [20].

In Finland, clinical trials of DMT for depression have included only adult patients. A randomized multicenter study [22] included 109 patients with depression from psychiatric outpatient clinics, occupational healthcare, and health centers. Participants were randomized into groups receiving DMT combined with treatment as usual (TAU) and TAU only. Depression symptoms were measured using the Beck Depression Inventory-21 (BDI-21), Clinical Outcomes in Routine Evaluation-Outcome Measure (CORE-OM), and Symptom Checklist-90 (SCL-90) questionnaires. A clinically significant decrease in depression symptom scores was observed in the DMT group compared with the control group [21].

## Aim of the study and hypotheses

The aim of this clinical trial is to investigate the effectiveness of a group-based dance-movement therapy in treating depression among adolescents. We tailored a group-based DMT treatment for adolescents based on knowledge from the multi-center study [21] of adults. The study protocol was registrated at ClinicalTrials.gov. (Table 1) 

Research questions are as follows:

1. Is dance movement therapy (DMT) effective in the treatment of adolescent depression?

2. Does the effectiveness of DMT persist for a 3-month follow-up period?
3. Are there clinical or background moderators that affect the treatment outcome?
4. What type of body image is observed in adolescents with depression, and does this body image change during DMT?.

Hypotheses related to the research questions are as follows:

1. Dance Movement Therapy (DMT) combined with treatment as usual (TAU) is more effective in treating adolescent depression than usual treatment alone
2. Treatment gains from active DMT persist until the 3-month follow-up.
3. The severity and recurrence of depression and comorbid psychiatric disorders predict poorer outcomes for DMT.
4. The body image of adolescents with depression is negative and will change to a more positive perception during DMT.

**Table 1:** Trial registration

Trial registration	
Study identification:	Protocol ID: HUS 391/2021, NCT: 07293806 Title: Dance movement therapy in the treatment of adolescent depression
Study status:	Recruiting
Sponsors/collaborators:	Collaborators: University of Helsinki, Helsinki University hospital (HUS) Responsible party: Primary investigator Riikka Valjakka, MD
Study description:	The aim of the trial is to measure effectiveness of DMT in adolescent depression
Conditions:	Depression
Study design:	Study type: Interventional Primary purpose: Treatment Interventional study model: Parallel assignement Number of arms: 2 Masking: None Allocation: Randomised Enrollment: 140
Outcome measures:	Beck Depression Inventory (BDI-21) YP-core ADRS Body image scale
Eligibility:	Minimum age: 13 Years Maximum age: 17 Years Sex: All Gender based: No Accepts healthy volunteers: No Criteria: Inclusion and exclusion criteria (see Table 2)
Contacts:	Central contact person: Riikka Valjakka, MD riikka.valjakka@hus.fi Study officials: Henna Haravuori, PhD, docent, Finnish Institute for Health and Welfare Mauri Marttunen, prof.emer. University of Helsinki

**Table 2:** Inclusion and exclusion criteria

Inclusion and exclusion criteria	
**Inclusion**:	**Exclusion**:
Age: 13–17 years Diagnosis: Moderate or severe depression ICD-10 codes F32.10, F32.11, F32.2, F33.10, F33.11 or F33.2* Measurements: BDI-21 > 16points (all participants) K-SADS interview > 4 criteria of severe depression (if accessable) *Patient may have other diagnoses besides depression	Acute psychotic episode Psychotic depression Personality disorder Severe anorexia Substance use disorder that require treatment PTSD with severe dissosiation Acute episodes of self injury Remission of depression, BDI-21 decrease over 50% Long term psychotherapy given by psychotherapist Psychophysical physiotherapy

## Methods

### Participants and recruitment

The aim is to recruit 140 adolescent patients with depression into the study. Recruitment began at Helsinki University Hospital’s adolescent outpatient unit in September 2022 (specialist level, Tier 3). Since September 2024, recruitment has been expanded to adolescent mental health services in Helsinki (targeted level, Tier 2). Patients’ ages range from 13 to 17 years. A clinical diagnosis of moderate or severe depressive disorder (ICD-10 codes F32.1x, F32.2, F33.1x, F33.2) is present, and comorbid illnesses may also be present. The requirement for the K-SADS-PL interview is that 4 or more depression symptom criteria must be fulfilled [22]. The K-SADS-PL interview is widely used at the specialist level; therefore, not all participants have been assessed with it.

Exclusion criteria include primary psychotic disorders, personality disorders, severe eating disorders, or substance use disorders requiring treatment. Patients receiving long-term psychotherapy administered by a psychotherapist are excluded; however, those receiving brief therapy (7–12 sessions) administered by a psychiatric nurse or psychologist are eligible. Patients receiving psychophysiological physiotherapy are also excluded, as the treatment outcome could be confounded by another body-oriented therapy (Table 2).

Patients meeting the inclusion criteria and their caregivers are informed about DMT and the trial during routine visits at the outpatient clinic. Those wishing to participate are screened and are required to sign written informed consent. Participants are divided into two age categories, with separate DMT groups for those aged 13–15 years and 16–17 years, to ensure developmental consistency within groups. Participants are randomly allocated into either the intervention or control group, stratified by age and sex. Randomization is conducted by a research nurse through drawing lots once two patients have been recruited from the same age and sex category. It may take time to randomize and assemble a group of five to six participants. There is no masking for the researcher or the staff responsible for gathering measurements.

The intervention group (DMT + TAU) will participate in the DMT group in addition to receiving treatment as usual (TAU). The control group will receive TAU.

### Intervention: dance-movement therapy group

The dance-movement therapy (DMT) intervention group is led by a dance-movement therapist, accompanied by an assistant leader and staff from adolescent mental health services, such as a nurse, psychologist, medical doctor, or occupational therapist, since autumn 2023. Each group consists of 5 to 6 patients.

Before the dance movement therapy (DMT) group begins, each participant will meet individually with the therapist to facilitate understanding of special needs and expectations. The purpose of this interview is also to share information and rules of the group therapy and to assess participants’ body image. Additionally, the importance of attending each session will be emphasized by the therapist.

DMT group sessions are conducted once a week for 12 weeks, with each session lasting 75 min. Sessions are held in the physical education room of the psychiatric hospital.

DMT therapists are professional health care or social workers who possess professional qualifications as dance-movement therapists. All therapists have experience in mental health work and youth engagement. Additional training in group-form dance movement therapy for the treatment of depression has been completed by the therapists. Prior to initiating the DMT groups, the therapists underwent training for a specially tailored intervention specific to this study. The training aimed to ensure consistency and quality of the therapeutic intervention across all groups. Therapists were instructed in group therapy methods appropriate for this patient population, based on knowledge acquired from previous studies. The training was conducted by a PhD psychologist and DMT therapist (PP), and a physiotherapist, DMT therapist, and supervisor (ML).

Therapists participate in a supervision hour four times during the 12-week therapy period under the guidance of a supervisor. A report is written by the therapist for each patient after the completion of therapy, which can be considered in planning further patient care.

The goal of this dance movement therapy (DMT) intervention is to examine participants’ relationships with their own bodies and movement in a safe, nonjudgmental atmosphere. Participants learn to observe their physical reactions, thoughts, and emotions. The aim is to strengthen a kind, curious, safe, and positive relationship with their own bodies, with attention to bodily activity and individual needs. Attendees learn various methods to modulate their feelings through embodied and movement-based techniques by engaging in mindful and communication-related physical exercises. The experiences of these exercises are communicated through discussion, drawing, and writing. The foundational principles of the group include trustworthiness, nonjudgmental attitudes, avoidance of harm (to oneself or others), and appreciation of embodied experiences. Each session begins with a warm-up exercise, followed by movement explorations within a specific theme that includes psychoeducation, and concludes with sharing experiences. Materials used in DMT include mattresses, balls, scarves, blankets, and beanbags. Suitable exercises for each group are selected by the therapist, who actively demonstrates the exercises with the group. The schedule and themes of each session are described in Table 3.

**Table 3:** Dance-movement therapy (DMT) treatment protocol

DMT treatment protocol			
	Theme	Exercises	Psychoeducation
1	Starting the group. Introduction. Discussing the rules of the group	Little movement warm-up, focus in pheripheral movement, using props to increase interest in movement. Breathing exercise. Short relaxation	Becoming part of a group. Rules and safety of the group. Emotions in the body
2	Getting familiar with the space and surroundings in the therapy room	Exploring the space through movement. Marking personal space with the help of props and making a dance in that. Therapist demonstrates the movements Breathing exercise	The importance of peer support. Encouragement for movement and expression
3	Safety, agency and sensing. Learning to know how it feels to be safe in the body	Getting feeling of safety by support and being rooted in the ground. Exploring different movement qualities in own kinesphere. Moving with a bean bag to sense the weight and the body surface	Interoseption and sensing the environment. Sympathetic and parasympathetic nervous system
4	Safety, activeness and options	Directions in the body and space, how to move in different levels. Exploring how movement can be initiated from different body parts. Finding different qualities in walking. Creating safety with breathing and nurturing self-touch on diaphragm	Self-care
5	Relationships	Attention to self/attention to others and surroundings. Co-operation with others by finding a common rhythm in movement.	Developing skills to observe and be aware of what happens in interaction.
6	Stress reduction	Observing the state of alertness of the body. Activating and relaxing the body by movement and images.	Detecting autonomic nervous system functions in the body
7	Breathing, senses, feelings and emotions.	Exploring how one relates with familiar, unfamiliar or pleasant/unpleasant movements; what they are for one-self. Cultivating acceptance towards emotions.	Getting familiar with emotions in the body
8	Caring about self and others.	Learning to respect the right to choose for one-self, when own body can be touched. Touching own body and self-massaging to bring ease to the body.	Safe and kind touch
9	Boundaries and distances. Personal space	Modulating and choosing distances: getting close and far from others. Mirroring movements. A dance of friendship	Communication of own boundaries and needs.
10	The positive side of self. Acceptance	How to communicate empathy to self by movement and rest?	What are the strengths in one-self?
11	Own needs and empathy towards self	Exploring empathetic movement. What do I need - rest, movement or care? What would support feelings of satisfaction?	How do I treat myself Self-compassion
12	The end of the therapy	Creating a piece of art together	What have I learned? Past–present - future

### Control group: treatment as usual

**Fig. 1:**
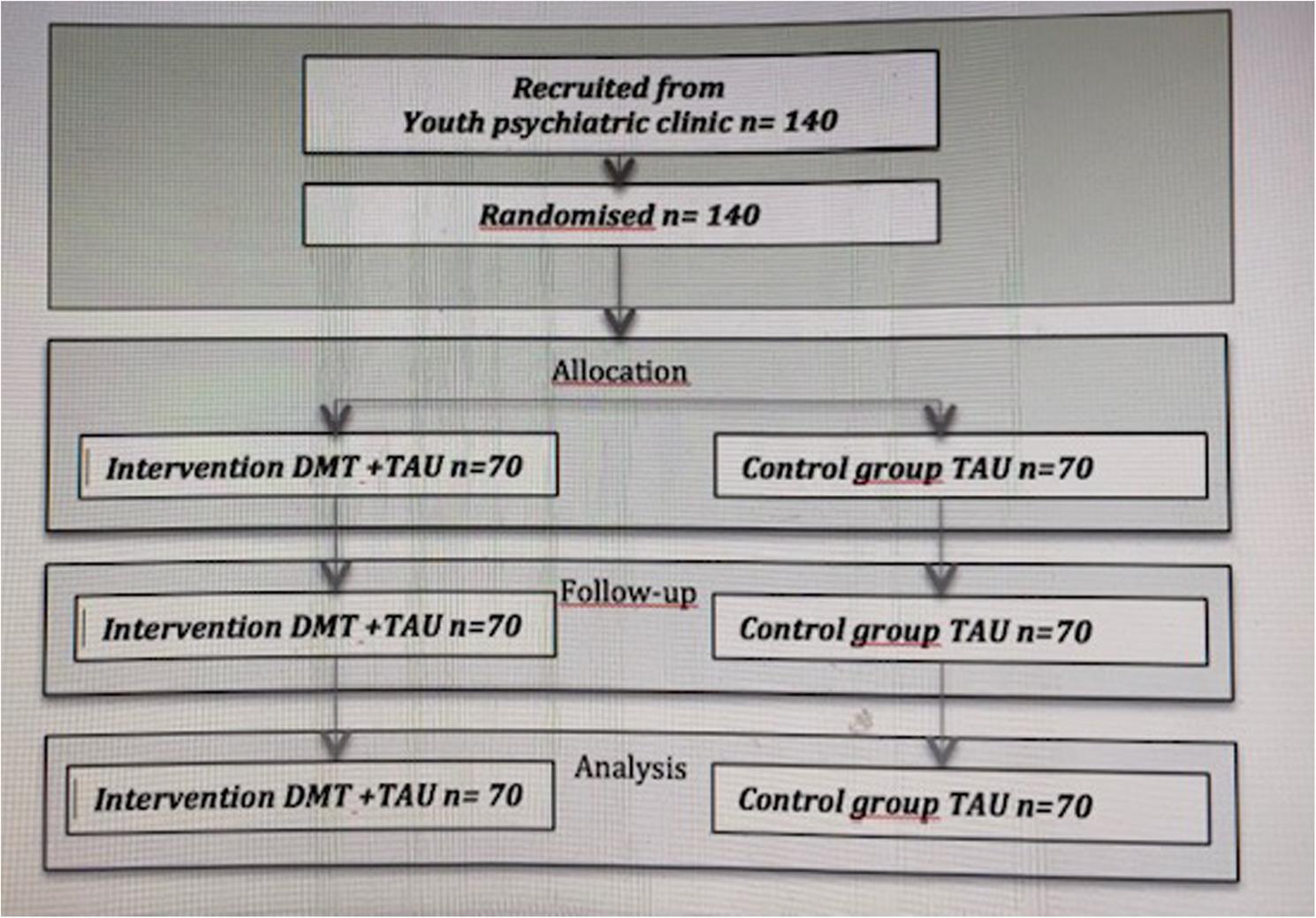
Flow chart

Members of the control group continue their treatment in the psychiatric outpatient clinic as previously planned. Most participants in the control group attend weekly visits to the clinic, which consist of psychoeducation, brief cognitive behavioral therapy, family counseling, or non-directive supportive care according to individual treatment plans. Medication is expected for many participants. Additionally, a few participants are on the waiting list for special therapy or rehabilitation and do not have regular clinic visits. Details of the treatment modalities and frequencies are being collected and will be described in future research reports. Patient flow is described in the flow chart (Fig. 1).

### Measures

Inclusion criteria include a clinical diagnosis of depressive disorder and a total score of 16 or more on the Beck Depression Inventory (BDI-21). The BDI-21 consists of 21 questions, with each question scored from 0 to 3 points, resulting in a total possible score of 0 to 63 points. Higher total scores on the scale indicate more severe depressive symptoms [23, 24]. 

### Primary outcome measures

The severity of depressive symptoms among participants will be assessed primarily using the Beck Depression Inventory-21 (BDI-21) and the Young Person’s Clinical Outcomes in Routine Evaluation (YP-CORE) questionnaires. The YP-CORE includes 10 questions, each scored from 0 to 4 points, with a total score range of 0 to 40 points. A higher total score on the scale indicates more severe psychological distress. The scale is specifically designed for monitoring changes in psychological distress [25]. 

Primary outcome measures will be assessed at baseline (before randomization) and at 8 weeks, 12 weeks, and 6 months from beginning of the intervention. As it may take time to randomize and assemble a group of five to six participants, variability in waiting times from baseline to intervention are reported. Both measures have been translated into Finnish, validated, and used in previous studies.

### Secondary outcome measures

The Adolescent Depression Rating Scale (ADRS) interview will be used as a secondary outcome measure. The ADRS interview consists of 10 questions, each scored from 0 to 6 points, for a total of 0 to 60 points. A higher total score indicates more severe depressive symptoms and impaired agency [26]. The ADRS interview will be conducted by clinical staff or a researcher who is unmasked to the treatment allocation.

The body image of the participants will be assessed using the Body Image Scale, a seven-point Likert scale consisting of 11 statements, such as “I feel comfortable in my body.” The Body Image Likert statements were developed from the Body Image Assessment Interview in a DMT study on depression [27]. This measure has primarily been used with adults [28]. It is offered to the youth in this study because it is concise and is proposed to consist of four factors: (1) memories of suffering, (2) ignorance of body sensations, (3) using the body for safe self-regulation, and (4) relating to one’s body in a comfortable way.

Secondary outcome measures will be assessed at baseline, 12 weeks, and 6 months from the beginning of the intervention.

### Moderators

Data are collected from patient records during the treatment period according to health register information systems and notes from psychiatric clinics. The onset, duration, and recurrence of depression were obtained from K-SADS-PL interviews or clinical records. Additionally, information about other measurement tools, psychiatric medication, living conditions, and education will be gathered. Living conditions are categorized as living with both parents, living with one parent, or other, which includes all other alternatives. Patient education is categorized as attainment of expected education or assignment to special support or a small group. No register information was available regarding the education of parents or their socioeconomic class.

### Sample size and power calculations

The appropriate sample size of 140 patients was determined by power calculation. It was estimated that the difference between the intervention and control groups using the specified measures is approximately 0.5 SD. With the test power set at 0.8 and a significance level of 0.05, calculations indicated that 64 participants would be needed for each group. If the treatment outcome by categorical variable is estimated to emerge in approximately 35% of the controls and 60% of the DMT group, 62 participants would be required for each group. Considering an expected 10% dropout rate, the target sample size should be 140.

### Data management

The collected data will be saved in the database in a categorized form.

### Statistical analysis

The intention-to-treat (ITT) sample will include all patients who have provided consent and are randomized. The per-protocol sample will include all patients from the control group and those from the DMT group who attended at least seven DMT group sessions. No more than 20% of missing items per scale are allowed and will be replaced by the mean value of the scores. Appropriate imputation methods will be selected for other missing data.

The primary outcomes are the time-by-treatment interactions in linear regression models with the ITT and per-protocol samples at the end of the intervention and at follow-up, measured by the Beck Depression Inventory-21 and the Young Person’s Clinical Outcomes in Routine Evaluation. Effect sizes of the differences in symptom scores within and between groups will be measured. The statistical significance level will be set at a p-value of < 0.05, and 95% confidence intervals will be calculated.

### Study status

Currently, 82 patients have been recruited, and 41 patients have participated in the DMT group. A total of 8 intervention groups have been conducted.

Recruitment will continue until 28.2.2026, and the DMT groups will continue until June 2026. The final measurements will be completed in September 2026, and data analysis will commence thereafter. Reporting of the findings is scheduled to begin in 2027.

## Discussion

Normal adolescent development includes belonging to a peer group. Mental disorders often cause delays in psychological development, which can lead to young patients experiencing social isolation. Participation in a therapy group offers peer support, which can help alleviate loneliness and facilitate social integration.

Creativity, art, and exercise may be beneficial in the recovery from depression. Young individuals may lack motivation for psychotherapy, and verbal communication may be more challenging compared to adults. Emotional instability and stress can create obstacles in verbal communication. By stabilizing the autonomic nervous system, dance movement therapy may assist young patients with these challenges.

There are some limitations to this trial. Patients are unmasked to the researchers because it is not feasible to mask attendance at a group therapy session, given that researchers work in the same institution. Recruitment of patients for the group proceeds slowly. Many patients with depression also experience social anxiety and are hesitant to participate in group therapies. Most patients are inactive and fatigued, making them uninterested in therapies that include physical activity; instead, they prefer individual talking therapy.

Previous studies indicate that the body image of patients with depression is negative, and these patients often neglect the physical reactions and feelings of their bodies. Improving the ability of young patients to sense and detect reactions and feelings within their own bodies may be beneficial.

This trial may provide new evidence-based treatment options for young patients with depression. The findings can be utilized in planning psychosocial treatments for adolescents, which may be implemented in psychiatric clinics, health centers, or school health care. A variation in therapy methods and treatment selection can assist clinics in expanding the range of therapies. This approach makes psychiatric care more accessible to patients, potentially accelerating recovery from depression. Novel treatment methods may also help prevent further episodes and chronic depression. It is crucial to treat adolescent depression effectively to prevent young people from dropping out of school and society.

## Data Availability

Data are available only upon request from the researcher. The data are not openly accessible due to ethical and legal restrictions associated with this highly sensitive data set containing patient information of minors. However, for meta-analytical and similar purposes, descriptive information and mean values may be provided upon request to the lead author.

## Abbreviations

DMT: Dance movement therapy
TAU: Treatment as usual
